# Cat fleas (*Ctenocephalides felis* clade ‘Sydney’) are dominant fleas on dogs and cats in New South Wales, Australia: Presence of flea-borne *Rickettsia felis, Bartonella* spp. but absence of *Coxiella burnetii* DNA

**DOI:** 10.1016/j.crpvbd.2021.100045

**Published:** 2021-07-30

**Authors:** Holly Hai Huai Huang, Rosemonde Isabella Power, Karen O. Mathews, Gemma C. Ma, Katrina L. Bosward, Jan Šlapeta

**Affiliations:** Sydney School of Veterinary Science, Faculty of Science, University of Sydney, Sydney 2006, Australia

**Keywords:** *Bartonella*, Illumina, Co-infection, Real-time PCR, *Rickettsia*, *cox*1, Q fever

## Abstract

The cat flea (*Ctenocephalides felis*) is the most common flea species parasitising both domestic cats and dogs globally. Fleas are known vectors of zoonotic pathogens such as vector-borne *Rickettsia* spp. and *Bartonella* spp. and could theoretically transmit *Coxiella burnetii*, the causative agent of Q fever. A total of 107 fleas were collected from 21 cats and 14 dogs in veterinary clinics, a feline rescue organisation and a grooming salon in New South Wales, Australia, to undergo PCR detection of *Bartonella* spp., *Rickettsia* spp. and *C. burnetii* DNA. Morphological identification confirmed that the cat flea (*C. felis*) is the most common flea in New South Wales, Australia, with only a single stick fast flea, *Echidnophaga gallinacea* recorded. The examined fleas (*n* = 35) at the *cox*1 locus revealed five closely related *C. felis* haplotypes (inter-haplotype distance < 0.5%). Multiplex TaqMan qPCR targeting the *gltA* (*Rickettsia* spp.) and *ssrA* (*Bartonella* spp.) genes was positive in 22.9% (95% CI: 11.8–39.3%) and 11.4% (95% CI: 3.9–26.6%) of samples, respectively. None of the DNA isolated from fleas was positive on TaqMan qPCRs targeting the *C. burnetii* IS*1111*, *Com*1 and *htp*AB genes. Co-infection of *C. felis* with *Bartonella henselae* and *Bartonella clarridgeiae* was demonstrated using *gltA* and *ssrA* Illumina next-generation amplicon sequencing. These findings reinforce the importance of flea control on domestic dogs and cats to effectively control the transmission of *Rickettsia felis* and *Bartonella* spp. The flea, however, is unlikely to be a vector of *C. burnetii* between companion animals and humans.

## Highlights

• The cat flea (*Ctenocephalides felis*) is the flea species on cats and dogs in New South Wales Australia.
• Absence of *Coxiella burnetii* DNA in flea extract, but presence of *Rickettsia felis.*
• Detection of *Bartonella* DNA using *gltA* and *ssrA* Illumina next-generation amplicon sequencing.

## Graphical abstract

**Figure:**
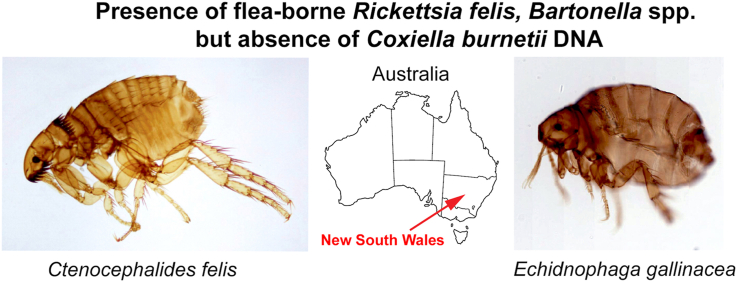

## 1 Introduction

The role of companion animal fleas in the epidemiology of *Bartonella henselae* – the causative agent of cat scratch disease – is well documented (Klotz et al., 2011). However, the exact role the flea plays in the transmission of other vector-borne zoonotic pathogens remains undefined (*Rickettsia felis*) or unknown (*Coxiella burnetii*) (Schriefer et al., 1994; Cutler et al., 2010; Klotz et al., 2011; Abdad et al., 2011; Toman et al., 2012; Njeru et al., 2016; Ng-Nguyen et al., 2020). Recently, companion dogs and cats were implicated in the transmission of both latter pathogens in Australia, including a Q fever outbreak (caused by *C. burnetii*) in a small animal veterinary hospital and a cluster of five probable cases of human infection with *R. felis* (Kopecny et al., 2013; Williams et al., 2011).

A member of the family *Rickettsiaceae*, *R. felis* causes flea-borne spotted fever (FBSF) (Abdad et al., 2011). In humans, clinical manifestations include signs of pyrexia, headaches, maculopapular rash, myalgias and eschar (Schriefer et al., 1994; Abdad et al., 2011). Over 30 arthropod species are recognised as potential vectors, but the cat flea (*Ctenocephalides felis*) is considered the main reservoir and vector. Transmission is believed to occur when an infected flea bites or contaminates open wounds with its faecal matter (Legendre and Macaluso, 2017). In Australia, *C. felis* is the dominant flea species on domestic cats and dogs (Šlapeta et al., 2011), hosting *R. felis* across the east coast at a prevalence of 6.7–19.8% (Barrs et al., 2010; Teoh et al., 2018).

The role of the cat flea (*C. felis*) in the transmission of *C. burnetii* is unknown. Q fever in humans is asymptomatic in approximately 60% of cases (Toman et al., 2012). Clinical disease manifests as an acute flu-like syndrome with non-specific signs of chills, malaise, sweating or fatigue (Raoult et al., 2000). Individuals with compromised cardiovascular function or endothelial cell defects can develop persistent focal diseases such as endocarditis or vascular infection (Raoult et al., 2000; Kampschreur et al., 2014). In addition, a debilitating chronic fatigue syndrome is experienced by at least 20% of acute Q fever patients (Morroy et al., 2016). In adults of most other mammalian species, coxiellosis is subclinical; however, abortions, infertility, stillbirth and weak progeny have occasionally been associated with the disease, especially in domestic ruminants, the primary source of human infections (Sanford et al., 1994; Eibach et al., 2012). Infection occurs primarily through inhalation of aerosolised infected materials. Arthropod transmission is theoretically possible, although an uncommon route for *C. burnetii* (Porter et al., 2011; Duron et al., 2015). Little is known about the role fleas, such as *C. felis*, play in *C. burnetii* transmission (Loftis et al., 2006; Psaroulaki et al., 2014; Špitalská et al., 2015; Kamani et al., 2015). No association has been found between the presence of fleas and seropositivity to *C. burnetti* in dogs and cats (Ma et al., 2020).

The cat flea (*C. felis*) in Australia is distributed along the coastal regions with three genetically distinct clades (Crkvencic and Šlapeta, 2019). The clade ‘Sydney’ of *C. felis* is the only species previously detected in New South Wales and in cooler regions of southern Australia, while the clade ‘Cairns’ and ‘Darwin’ display preponderance in tropical northern Australia and may also be found further south due to climate change (Crkvencic and Šlapeta, 2019). The capacity of these genetic clades to carry zoonotic pathogens and those that exist globally within *C. felis* is not yet known (Lawrence et al., 2019).

The aim of this study was to confirm the presence of *C. felis* on dogs and cats and evaluate their role in carriage of DNA of selected zoonotic vector-borne diseases. In an effort to shed more light on the vector contribution of fleas towards Q fever and FBSF, we applied qPCR diagnostic assays to detect *C. burnetii*, *R. felis* and *Bartonella* spp. in fleas collected from dogs and cats visiting veterinary clinics, a rescue organisation and a grooming salon.

## 2 Materials and methods

### 2.1 Flea collection

From November 2019 to May 2020 fleas were collected from around New South Wales, Australia (Table 1). Fleas were collected opportunistically from dogs and cats by veterinarians, veterinary nurses, groomers and shelter carers as part of their routine work. Fleas were placed in 70% ethanol and stored at room temperature before being transferred into the freezer (−20 °C).

**Table 1:** Sequence and product lengths of target gene primers for *Coxiella burnetii* qPCR

Primer	Primer sequence (5′-3′)	Product length (bp)	Final concentration (nM)	Reference
**IS*1111***^a^		146		de Bruin et al. (2011)
Forward primer	CGCAGCACGTCAAACCG		300	
Reverse primer	TATCTTTAACAGCGCTTGAACGTC		300	
Probe	FAM-ATGTCAAAAGTAACAAGAATGATCGTAAC-BHQ1		200	
***groEL***^b^		114		Bond et al. (2016)
Forward primer	GTGGCTTCGCGTACATCAGA		300	
Reverse primer	CATGGGGTTCATTCCAGCA		300	
Probe	CFO560-AGCCAGTACGGTCGCTGTTGTGGT-BHQ1		200	
***com1***^c^		76		Lockhart et al. (2011)
Forward primer	AAAACCTCCGCGTTGTCTTCA		400	
Reverse primer	GCTAATGATACTTTGGCAGCGTATTG		400	
Probe	Quasar670-AGAACTGCCCATTTTTGGCGGCCA-BHQ2		200	

*Note*: FAM, 6-Carboxyfluorescein; BHQ1, Black Hole Quencher-1; CAF560, CAL Flour Orange 560 Amidite; Quasar670, Quasar 670 Carboxylic Acid; BHQ2, Black Hole Quencher-2.

a Insertion sequence 1111 (IS*1111*).

b Heat-shock operon (*htp*AB).

c Outer membrane protein (*com1*).

### 2.2 Morphological and molecular identification using cytochrome *c* oxidase subunit 1

All fleas were examined individually under a microscope (5–200×, Olympus, Australia) and placed to a genus and species as previously described (Dunnet and Nardon, 1974; Lawrence et al., 2019). A minimum of one flea of each flea species (if more than one species was present or if the species was equivocal on morphological examination) from each animal was selected for molecular characterisation. Thirty-five fleas were incised on the dorsal caudal abdomen using a sterile scalpel blade before being placed in a microcentrifuge tube (Eppendorf, Macquarie Park, Australia) which was then incubated in a heat block set at 70 °C for 15 min to evaporate the ethanol in which they were stored (Lawrence et al., 2015). Flea DNA was extracted using the Isolate II Genomic DNA kit (BioLine, Eveleigh, Australia) according to the manufacturer's protocol with DNA eluted in 100 μl of elution buffer and stored at −20 °C. For each batch of DNA extractions, an extraction with no flea was included and the eluate served as a non-template control (negative extraction control, NEC).

Extracted flea DNA samples were subjected to conventional polymerase chain reaction (PCR) targeting cytochrome *c* oxidase subunit 1 (*cox*1) as previously described (Lawrence et al., 2015, 2019). Polymerase chain reaction amplification was performed in a 30 μl reaction mixture containing 15 μl MyTaq Red Mix (BioLine), with 2 μl DNA and nuclease-free water. Assays were performed in a T100 cycler (Bio-Rad, Australia) with an initial denaturation at 95 °C for one minute followed by 35 cycles of 95 °C for 15 s, 55 °C for 15 s, 72 °C for 10 s, and a final elongation for 5 min at 72 °C. All reactions were run with a NEC and sterile PCR water in place of DNA (nontarget control, NTC). Amplicons of *cox*1 were sequenced (Macrogen Ltd, Seoul, Korea) and DNA sequences were assembled using CLC Main Workbench 21 (CLC bio, Qiagen, Chadstone, Australia) and compared to reference *cox*1 haplotypes (h1-h90) *sensu* Lawrence et al. (2019) and associated with the three clades (‘Sydney’, ‘Cairns’, ‘Darwin’) *sensu* Crkvencic and Šlapeta (2019).

### 2.3 Molecular detection and identification of vector-borne pathogens including *Rickettsia* spp., *Bartonella* spp. and *C. burnetii*

An aliquot of flea DNA underwent multiplex TaqMan qPCR targeting the *C. burnetii* multicopy insertion sequence gene IS*1111* (146-bp amplicon), and two single copy genes: *com1* (76-bp amplicon) the outer membrane protein-coding gene, and *groEL* (114-bp amplicon; heat-shock operon; *htp*AB) (de Bruin et al., 2011; Lockhart et al., 2011; Bond et al., 2016). Each reaction contained 1× SensiFAST Probe No-ROX Kit (BioLine, Australia), primers and probe (Table 1), 2 μl DNA and nuclease-free water in a total volume of 10 μl. Duplicate assays were performed using a Bio-Rad-CFX Real-Time PCR Thermocycler (Bio-Rad Laboratories Pty Ltd, Gladesville, NSW, Australia) with an initial denaturation at 95 °C for 3 min followed by 40 cycles of denaturation at 95 °C for 10 s and annealing at 60 °C for 40 s. Each qPCR run included a NTC (with nuclease-free water in place of DNA), and positive control *C. burnetii* DNA of known copy number (Amplirun® Vircell, Granada, Spain) at 1100, 550 and 25 copies of the *C. burnetii* genome per reaction corresponding to a quantification threshold (C_t_) of ~34 for IS*1111* and a C_t_ of ~36 for both *com*1 and *gro*EL. Samples were considered positive if amplification occurred for all three genes at or below the C_t_ for each gene.

A TaqMan probe-based real-time PCR for *Rickettsia* and *Bartonella* species targeting the citrate synthase (~75-bp amplicon; *gltA*) gene and a transfer-messenger RNA (~300-bp amplicon; *ssrA*) respectively, was used to screen DNA from individual fleas for vector-borne pathogens (Stenos et al., 2005; Diaz et al., 2012). The reactions were performed in duplicate using the CFX96 Touch^TM^ Real-Time PCR Detection System (BioRad, Australia) and contained 1× SensiFAST Probe No-ROX Kit (BioLine, Australia), primers and probes, 2 μl template DNA in a total volume of 20 μl. Data were analysed using the corresponding CFX Maestro 1.0 software (BioRad, Australia) as previously described (Šlapeta and Šlapeta, 2016). Each run included positive and negative controls (NTC, NEC). Results were considered positive if duplicates yielded C_t_ values < 36. Suspect positive results were determined when one or more repeats yielded C_t_ values ≥ 36 and samples were considered negative if neither repeat crossed the threshold (C_t_ > 40). Positive *Bartonella* results were sent to Macrogen for sequencing (Macrogen Ltd, Seoul, South Korea). Samples considered either positive or suspect positive for *Rickettsia* spp. (C_t_ value < 38) were further identified using a pair of conventional nested PCRs targeting the outer membrane protein A (*ompA*) gene and *gltA* (Stenos et al., 2005; Šlapeta and Šlapeta, 2016). PCR products were sequenced at Macrogen Inc. (Seoul, Korea) and assembled using CLC Main Workbench 21 (CLC bio, Qiagen, Australia).

Illumina overhang adapters were added to two sets of amplicon primers to amplify a ~301-bp and ~360-bp region of the *ssrA* and *gltA* genes of *Bartonella*, respectively (Power et al., 2021). Next-generation sequencing (NGS) of first stage PCRs were performed in-house according to the 16S Metagenomic Sequencing Library Preparation instructions for the Illumina MiSeq System (Illumina, Australia). The *ssrA* and *gltA* conventional PCR reactions were performed with Q5 Hot Start High-Fidelity 2X Master Mix (New England Biolabs, Australia). The protocol for each *Bartonella* sample varied according to their starting DNA concentrations. The PCR products were visualized on a 2% agarose gel with the aid of GelRed to ensure the presence of PCR products of the required size. The *ssrA* and *gltA* PCR amplicons were submitted for NGS at the Ramaciotti Centre for Genomics, University of New South Wales, Australia, for sample preparation and paired-end 250 bp Illumina sequencing on MiSeq system (Illumina, Australia). The DADA2 pipeline (package version 1.19.2; Callahan et al., 2016) was used to infer the exact *Bartonella* amplicon sequence variants (ASVs) from flea samples as previously described in Power et al. (2021). Local reference *Bartonella* databases were imported into DADA2 to classify the ASVs using two different classification methods; the ‘assignSpecies’ and ‘assignTaxonomy’ functions. ASVs that were not assigned to a gene and *Bartonella* species were manually removed. The *ssrA* and *gltA* reads were then tabulated separately and the proportions (%) of each ASV in each sample were calculated for both genes. To address the potential issue of misassigned reads, all ASVs with < 1% in a sample were manually removed and replaced with ‘0’. ASVs were visually inspected, and their species assignment confirmed in CLC Main Workbench 21 (CLC bio, Qiagen, Australia) and aligned to sequences from our local curated *Bartonella* reference databases (Power et al., 2021).

## 3 Results

A total of 107 fleas were collected opportunistically from 32 dogs and cats (HH-1 to HH-32) in New South Wales, Australia including a cat shelter, grooming salon, and veterinary clinics (Table 2). Most fleas (93.5%, 102/107) were morphologically identified as the cat flea (*C. felis*). Three *Ctenocephalides* sp. fleas had equivocal morphological characteristics due to being damaged or having an ambiguous second notch on the hind tibia between the apical and post-median setae. One flea (HH-21-2) from a dog from north-west New South Wales, Australia, was identified as the stick fast flea (*Echidnophaga gallinacea*). Overall, there were 33 males, 72 females, one *C. felis* whose sex was not possible to determine and one male *E. gallinacea.*

**Table 2:** Summary of flea material collected from dogs and cats in New South Wales, Australia

Animal category	No. in the category (%)	No. of fleas
Owned	16 (21%)	77
Greater Sydney	9 (17%)	53
Grooming salon	7	37
Canine	4	30
Feline	3	7
Veterinary clinic	2	16
Canine	1	12
Feline	1	4
North-west New South Wales	7 (29%)	24
Veterinary clinic	7	24
Canine	6	23
Feline	1	1
Stray	15 (58%)	26
Greater Sydney	15	26
Cat shelter	15	26
Feline	15	26
Unknown	3 (75%)	4
Greater Sydney	1	4
Shelter	1	4
Feline	1	4
Grand total	32	107

At least one *C. felis* from each animal was selected for DNA isolation including *C. felis* specimens with equivocal morphology, and the single *E. gallinacea* (*n* = 35, Table 2, Supplementary Table S1). The *cox*1 gene was successfully amplified and sequenced from DNA extracts of all specimens. There were five *cox*1 haplotypes of *C. felis* (Cf_h1-h5) and a single *E. gallinacea* haplotype (Eg_h1). The *cox*1 haplotype Cf_h1 was the most dominant with 26 representatives, the remaining haplotypes had 1–4 representatives. All three *Ctenocephalides* sp. fleas with equivocal morphology had a *cox*1 sequence that was identical to the *C. felis cox*1 haplotype Cf_h1. Haplotypes Cf_h1 to Cf_h5 differed from each other by single variable nucleotides. The *cox*1 sequence of *E. gallinacea* was 100% identical to the reference *cox*1 sequence from Australia (KT376440, Lawrence et al., 2015). The Cf_h1 of *C. felis* was identical to “h1” *sensu* Lawrence et al. (2019), and together with the remaining haplotypes represent the clade ‘Sydney’ *sensu* Crkvencic and Šlapeta (2019).

Multiplex TaqMan qPCR targeting the IS*1111*, *Com*1 and *htp*AB genes for *C. burnetii* was negative for all examined fleas (0/35, 95% CI: 0–11.8%). Multiplex TaqMan qPCR targeting the *gltA* (*Rickettsia* spp.) and *ssrA* (*Bartonella* spp.) was positive in 8 (8/35, 95% CI: 11.8–39.3%) and 4 (4/35, 95% CI: 3.9–26.6%) samples, respectively. In addition, 3 and 4 samples were considered suspect positive for amplification of *gltA* (*Rickettsia* spp.) and *ssrA* (*Bartonella* spp.), respectively, based on C_t_ values > 36 (Table 3, Table 4). All NTC and NEC reactions remained negative throughout this study.

**Table 3:** Summary of flea material used for molecular diagnostics for *Bartonella* and *Rickettsia*

Animal category	Negative	Positive	Suspect	Grand total
*Bartonella* sp. qPCR				
Greater Sydney	20	4	2	26
Owned	9		1	10
Stray	11	3	1	15
Unknown		1		1
North-west New South Wales	7		2	9
Owned	7		2	9
Grand total	27	4	4	35
*Rickettsia* spp. qPCR				
Greater Sydney	17	7	2	26
Owned	8	1	1	10
Stray	9	6		15
Unknown			1	1
North-west New South Wales	6	1	2	9
Owned	6	1	2	9
Grand total	23	8	4	35

**Table 4:** *Rickettsia* and *Bartonella* diagnostics on fleas from New South Wales, Australia

Sample	Flea species^a^	qPCR *Rickettsia*		Sanger *gltA*/*ompA*^b^	qPCR *Bartonella*		Illumina *ssrA* + *gltA*	Locality	Host	Age (years)	Sex	Ownership
		C_t_ value	Result		C_t_ value	Result						
HH-2-1	*C. felis*	38.62	Suspect			Negative		Greater Sydney	Feline	1–2	M	Owned
HH-5-1	*C. felis*	34.98	Positive		26.25	Positive	*B. henselae*	Greater Sydney	Feline	1	M	Stray
HH-6-1	*C. felis*	21.38	Positive	*R. felis* (100%)/*R. felis* (100%)		Negative		Greater Sydney	Canine	0.3	M	Owned
HH-13-1	*C. felis*	37.86	Suspect	*R. felis* (100%)/*R. felis* (100%)	34.66	Positive	*B. henselae*	Greater Sydney	Feline	0.2	F	Unknown
HH-14-1	*C. felis*	33.31	Positive	*R. felis* (100%)/*R. felis* (100%)	37.39	Suspect		Greater Sydney	Feline	0.3	M	Stray
HH-15-1	*C. felis*	21.41	Positive	*R. felis* (100%)/*R. felis* (100%)		Negative		Greater Sydney	Feline	0.3	F	Stray
HH-16-1	*C. felis*	19.82	Positive	*R. felis-*like (99%)/-	26.05	Positive	*B. henselae*	Greater Sydney	Feline	0.3	F	Stray
HH-18-1	*C. felis*	33.42	Positive	*-/R. felis* (100%)	18.41	Positive	*B. henselae* *B. clarridgeiae*	Greater Sydney	Feline	0.2	M	Stray
HH-19-1	*C. felis*		Negative		37.61	Suspect		North-west New South Wales	Feline	0.2	M	Owned
HH-21-1	*C. felis*	35.80	Positive			Negative		North-west New South Wales	Canine	1	M	Owned
HH-22-2	*C. felis*		Negative		37.66	Suspect		Greater Sydney	Canine	4	F	Owned
HH-23-1	*C. felis*	38.17	Suspect	*R. felis* (100%)/*R. felis* (100%)	38.32	Suspect		North-west New South Wales	Canine	0.4	M	Owned
HH-26-1	*C. felis*	39.74	Suspect			Negative		North-west New South Wales	Canine	5	F	Owned
HH-28-1	*C. felis*	37.52	Positive			Negative		Greater Sydney	Feline	0.2	M	Stray

a All *C. felis* were typed using *cox*1 as “Clade Sydney”.

b DNA amplification and sequencing, percent identity against reference genome of *R. felis* (CP000053).

Amplification and DNA sequencing of *Rickettsia*-positive and suspect samples with conventional nested PCR targeting *gltA* and *ompA* genes revealed five fleas (HH-6-1, HH-13-1, HH-14-1, HH-15-1, HH-23-1) that sequenced as identical (100%) DNA with *R. felis* reference *gltA* (Parola et al., 2003) and *ompA* (Ogata et al., 2005). HH-18-1 amplified only using *ompA* assay and its DNA sequence was identical with the *R. felis* reference. An additional sample (HH-16-1) that amplified only *gltA* yielded a sequence that was 99% identical to the *R. felis* reference DNA with a variation of one single nucleotide at the *gltA* gene (Table 4).

**Fig. 1:**
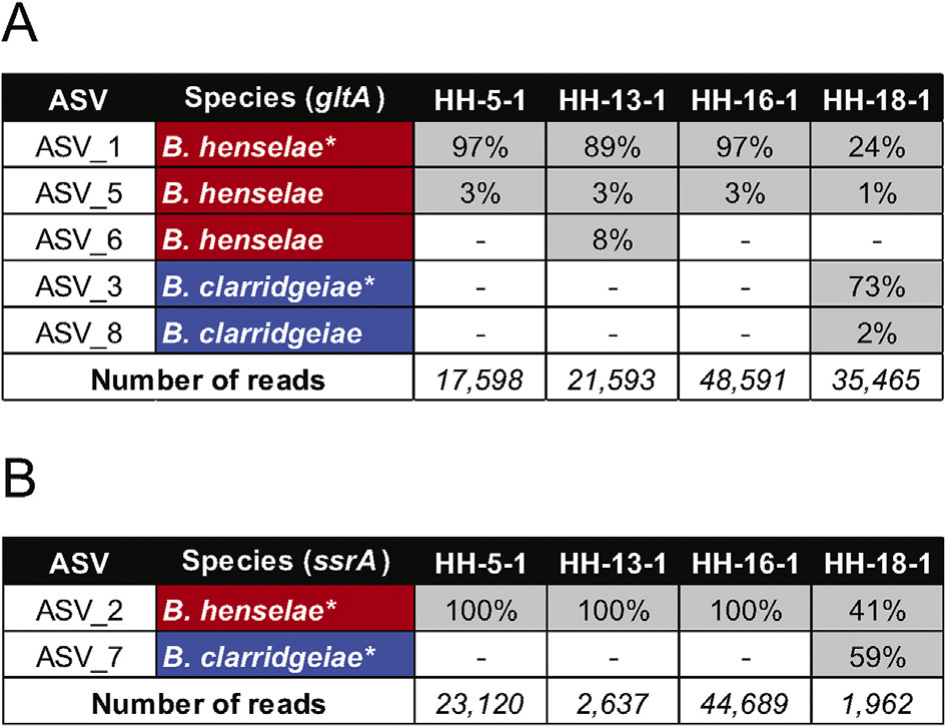
Amplicon next-generation sequencing results of flea DNA samples for the identification of *Bartonella* species. The proportion of amplicon sequence variants (ASVs) and the number of reads obtained from each flea sample for *gltA* (**A**) *ssrA* (**B**) amplicons are shown. The data were processed using DADA2 and perfect match to reference genomic sequence of *Bartonella* spp. is indicated by ‘∗’. At *gltA*, alternative ASVs were detected for both *Bartonella* spp. sequences that were 1–2 nucleotide different to its reference sequence.

Sanger DNA sequencing of the *ssrA* product failed to unambiguously resolve the *Bartonella ssrA* DNA sequence. We used conventional PCR with Illumina tagged *Bartonella-*specific *ssrA* and *gltA* primers to amplify these regions from DNA of seven *Bartonella* spp. positive and suspect positive samples. Amplification was successful for all four positive samples but was unsuccessful for the three suspect positive samples. The positive amplicons were subject to Illumina DNA sequencing and on average yielded 30,811 paired-end good quality sequences for *gltA* and 18,102 paired-end good quality sequences for *ssrA.* The HH-18-1 sample showed a mixed pattern because for both *gltA* and *ssrA*, it had sequence reads matching *B. henselae* (strain Houston-1) and *Bartonella clarridgeiae* (strain 73). At *ssrA*, 41% of the reads belonged to *B. henselae* and 59% to *B. clarridgeiae*. At *gltA*, 25% of the reads belonged to *B. henselae* and 75% to *B. clarridgeiae*. The remaining three samples had only *B. henselae* sequences (Table 4; Fig. 1).

## 4 Discussion

Arthropods are known vectors of zoonotic pathogens worldwide, the most common of which is the cat flea, *C. felis* (Clark et al., 2018; Lawrence et al., 2019), a well-documented carrier of *Rickettsia* and *Bartonella* species (Barrs et al., 2010; Šlapeta and Šlapeta, 2016). In this study the presence of *Rickettsia* and *Bartonella* species DNA and that of *C. burnetii*, the causative agent of Q fever, in fleas of cats and dogs were investigated in Greater Sydney and rural communities in New South Wales. All but one flea were identified to be *C. felis*, consistent with previous studies in Australian companion animals (Šlapeta et al., 2011; Lawrence et al., 2014). The only other flea species identified in this study was the stickfast flea (*E. gallinacea*), which is rarely found on dogs and cats, since it traditionally favours residence on avian species (Šlapeta et al., 2011). This study confirmed that only the clade ‘Sydney’ of *C. felis* is present in New South Wales (Šlapeta et al., 2011; Lawrence et al., 2015; Chandra et al., 2017; Crkvencic and Šlapeta, 2019). In neighbouring New Zealand, only the clade ‘Sydney’ of *C. felis* has been documented and a study by Chandra et al. (2017) found similar percentages of fleas positive for *Bartonella* and *Rickettsia* using multiplexed TaqMan qPCR on 38 *C. felis* DNA samples, with 5.3% (*n* = 2) positive for *Bartonella* and 18.4% (*n* = 7) positive for *Rickettsia* DNA. The identification of co-infection of *B. henselae* and *B. clarridgeiae* in a single flea was demonstrated using recently developed multi-locus Illumina next-generation amplicon sequencing, demonstrating the advantages that this technology and approach enable over conventional PCR alone (Power et al., 2021).

Since the discovery of *R. felis* in the 1990s, the organism has frequently been detected in *C. felis* (Adams et al., 1990; Legendre and Macaluso, 2017). Detection of *R. felis* DNA in arthropods ranges from ~1% to 100% across various countries (Reif and Macaluso, 2009). Our results of DNA detection of *R. felis* in fleas is similar to the 6.7–36.0% reported in other Australian studies (Schloderer et al., 2006; Barrs et al., 2010; Teoh et al., 2018) and 15.5% (Teoh et al., 2018) reported in coastal regions of New South Wales including the Central Coast, Northern Beaches, and Sydney. The cat flea (*C. felis*) likely plays an important role in the transmission of *R. felis* to humans, acting both as a vector and reservoir (Barrs et al., 2010; Hirunkanokpun et al., 2011; Legendre and Macaluso, 2017). The focus in Australia has been on fleas from coastal cities, presumably due to the importance of optimal temperatures and humidity for flea development in these geographical areas (Silverman and Rust, 1983; Metzger and Rust, 1997). *Rickettsia* *felis* is able to successfully maintain its population through both horizontal (Hirunkanokpun et al., 2011; Brown et al., 2015) and vertical transmission (Wedincamp and Foil, 2002) which might explain why densely populated areas in Australia have the highest incidence of human *R. felis* infection cases recorded (Teoh et al., 2016; Hii et al., 2017). Therefore, flea control in domestic animals is required to effectively reduce the transmission of *R. felis* to humans, and is especially important in densely populated areas.

*Coxiellaburnetii* was not detected in any of the fleas collected. In cats and dogs, *C. burnetii* seroprevalence varies regionally, being the highest in cattery confined breeding cats (Shapiro et al., 2015) and dogs from rural communities (Shapiro et al., 2016, 2017). By comparison, seropositivity in shelter or household Australian dogs and cats in urban areas are relatively low (Shapiro et al., 2015, Shapiro et al., 2016); however, seropositivity as high as 55.9% has been found in dogs in rural communities near a previous Q fever outbreak (Ma et al., 2020). Given that the majority of fleas in the present study were obtained from either owned or stray animals in metropolitan areas, the absence of positive *C. burnetii* DNA was not unexpected. Although transmission of *C. burnetii* is most often airborne (Porter et al., 2011), *C. burnetii* DNA has been detected in a multitude of tick species that parasitise dogs and cats (Cooper et al., 2013; Duron et al., 2015) and ticks are theoretically capable of transmitting the bacterium through secretion of contaminated faeces (Korner et al., 2020) or tick bites (Duron et al., 2015). This is the first study to investigate for evidence of *C. burnetii* DNA in fleas collected from cats and dogs in Australia. In agreement with our findings, *C. burnetii* DNA was not detected in fleas from Slovakia (Špitalská et al., 2015), Israel (Kamani et al., 2015) and Egypt (Loftis et al., 2006). In addition, Ma et al. (2020) found no association between the presence of fleas and seropositivity to *C. burnetii* in dogs or cats in Australia. The highest detection rate of *C. burnetii* DNA in fleas, however, has been reported in a study conducted in Cyprus which used a qPCR assay targeting the *C. burnetii* IS*1111* insertion sequence to detect *C. burnetii* in DNA extracts from pooled flea sets. This study reported an overall *C. burnetii* DNA detection rate of 16.3% (25 of the 153 flea pools tested were positive) and a species specific pooled prevalence of 38.1% in dog fleas (*Ctenocephalides canis*) from foxes, 16.6% in *C. felis* fleas from both foxes and rats and 10.7% in *Xenopsylla cheopis* fleas from rats (Psaroulaki et al., 2014). The IS*1111* is a commonly used target in qPCR due to its enhanced sensitivity of detection (Klee et al., 2006). Until quite recently *C. burnetii* was the only described species of its genus; however, it is now recognised that *Coxiella*-like endosymbiont bacteria, which are closely related to but genetically distinct from *C. burnetii*, are present in soft and hard ticks (Wilkinson et al., 2014). IS*1*111 has been demonstrated to be widespread in these *Coxiella*-like bacteria and is therefore not specific to *C. burnetii* (Duron, 2015). It is possible that *Coxiella*-like DNA may also exist in other arthropods such as fleas, and thus the sole use of IS*1111* as a diagnostic indicator may be misleading due to the potential false positive amplification of DNA from *Coxiella*-like endosymbionts. Furthermore, the existence of the IS*1111* insertion sequence in all strains of *C burnetii* has been a subject of recent debate (Marmion et al., 2005; Rolain and Raoult, 2005). Although the qPCR in the present study targeted the IS*1111* insertion sequence, the inclusion of the single copy genes *com1* and *groEL* in the qPCR assay increased the chances of detecting strain variants of *C. burnetii* that may not contain the IS*1111* insertion sequence and additionally minimised the likelihood of obtaining false positive results due to amplification of *Coxiella*-like DNA.

## 5 Conclusions

The cat flea (*C. felis*) was confirmed as the most common flea on dogs and cats in New South Wales, Australia, and there was an absence of *cox*1 clades other than clade ‘Sydney’. While DNA of the zoonotic pathogens *R. felis* and *Bartonella* spp. was demonstrated in fleas, *C. burnetii* DNA was not detected in this investigation, consistent with previous studies. A combination of molecular tools to characterise both the arthropods and the potential zoonotic pathogens enabled us to detect co-infection of *Bartonella* spp. and *R. felis*. Illumina next-generation amplicon sequencing was also applied to demonstrate co-infection of *B. henselae* and *B. clarridgeiae*.

## Funding

This research was in part funded by the Sydney School of Veterinary Science Research & Enquiry Unit of Study 2020 fund.

## CRediT author statement

Holly Hai Huai Huang: Conceptualization; Data curation; Formal analysis; Investigation; Methodology; Validation; Roles/Writing - original draft; Writing - review & editing. Rosemonde Isabella Power: Formal analysis; Methodology; Validation; Writing - review & editing. Karen Olivia Mathews: Investigation; Data curation; Methodology; Resources; Writing - review & editing. Gemma C. Ma: Investigation; Data curation; Methodology; Resources; Writing - review & editing. Katrina L. Bosward: Conceptualization; Funding acquisition; Investigation; Methodology; Project administration; Resources; Supervision; Writing - review & editing. Jan Šlapeta: Conceptualization; Data curation; Formal analysis; Funding acquisition; Investigation; Methodology; Project administration; Resources; Supervision; Validation; Writing - review & editing.

## Data availability

The nucleotide sequence data generated in this study were deposited in GenBank (NCBI) under the accession numbers MZ381608-MZ381642 and MZ420158-MZ420169. Raw fastq sequence data was deposited at SRA NCBI BioProject: PRJNA737164. Sequence data, associated supplementary and additional data are available at LabArchives (https://doi.org/10.25833/2c6t-2276).

## Declaration of competing interests

The authors declare that they have no known competing financial interests or personal relationships that could have appeared to influence the work reported in this paper.

## Appendix A Supplementary data

Multimedia component 1 **Supplementary Table S1.** Summary of samples and diagnostics. Multimedia component 1
